# The incidence of unexpected poor ovarian response in Chinese young women

**DOI:** 10.1097/MD.0000000000014379

**Published:** 2019-02-15

**Authors:** Jing Zhuang, Hengli Li, Xiaohong Li, Dongmei Tian, Dan Yang, Minghui Zhu

**Affiliations:** a School of Medical and Life Science/Reproductive & Women-Children Hospital, Chengdu University of Traditional Chinese Medicine; b The Second Hospital of West China, Sichuan University; National Center of Birth Defects Monitoring of China; National Office of MCH Surveillance; Chengdu, Sichuan Province, China.

**Keywords:** Chinese women, in vitro fertilization, incidence, intracytoplasmic, sperm injections, unexpected poor ovary response

## Abstract

Unexpected poor ovarian response (UPOR) is a problem for both clinicians and infertile couples, because our understanding of this situation is limited. This article investigated incidence of UPOR in women <35 years with normal ovarian reserve function with further analysis.

This is a retrospective study, which included 567 women who accepted their first IVF-ET/ICSI. Based on the number of oocytes retrieved, clinic pregnancy rate of fresh cycle, and cycle cancellation rate of fresh cycle, the included cycles were divided into three groups namely unexpected poor ovarian response (UPOR) (*n* = 120), for which number of oocytes retrieved was not more than 6; normal ovarian response (NOR) (*n* = 223), for which number of oocytes retrieved was between 7 and 12; and unexpected high ovarian response (UHOR) (*n* = 224), for which the number of oocytes retrieved was 13 at least. The comparisons of clinical outcomes and correlated hormones among groups were carried out.

The incidence of UPOR in Chinese women is 21.16%. Patient age (*χ*^2^ = 6.177, *P* = .0129), basic FSH (*χ*^2^ = 20.585, *P* < .0001), basic LH (*χ*^2^ = 11.689, *P* = .0006), and AFC (*χ*^2^ = 8.053, *P* = .0045) might be helpful for diagnosis of UPOR.

The basic evaluation of ovarian function may no longer be simplified into normal and abnormal ovarian reserve function; rather, by using a detailed numerical analysis, such as basal FSH and LH levels, the ovarian response to ovulation induction may be predicted to some extent.

## 1 Introduction

The adequate number of retrieved oocytes is an important prerequisite for the success of in vitro fertilization (IVF) treatment. As early as 2011, Sunkara SK et al^[1]^ indicated in their retrospective analysis of more than 400,000 cases of IVF cycles that the number of retrieved oocytes might be used as an independent predictor of the live birth rate of the treatment cycle; thus, the number of retrieved oocytes might directly determine the IVF treatment success rate.^[2]^

For infertile women >35 years, particularly those with apparent diminished ovarian reserve (DOR), physicians and the patients themselves have certain psychological expectations with regard to the number of retrieved oocytes. That is, physicians and patients generally concur and are adequately psychologically prepared with regard to the influence of too few retrieved oocytes in the assisted reproduction treatment.^[3–5]^ Therefore, both physicians and patients could accept the termination of assisted reproduction in cases of unavailable embryos due to an insufficient number of retrieved oocytes. Patients >35 years with poor ovarian responses to controlled ovarian hyperstimulation (COH) and too few retrieved oocytes are generally considered to have expected poor ovarian responses.^[6]^ However, for women <35 years with normal ovarian reserve function, both physicians and patients often lack adequate psychological preparation for a poor ovarian response, particularly in patients undergoing the assisted reproduction treatment (ART) for the first time, who may lack additional reference information with regard to the response to COH.^[7]^ Furthermore, such a response may cause other serious difficulties and increased psychological pressure for the couples. Therefore, patients with normal ovarian reserve below age 35 years with poor ovarian responses to COH and too few retrieved oocytes are considered to have unexpected poor ovarian responses (UPOR).

Currently, clinicians lack sufficient data to answer questions regarding the odds of an UPOR when they encounter the couples undergoing IVF/ICSI treatment for the first time and the wives have a normal ovarian reserve to accept a regular COH protocol; whether an UPOR realistically affects the therapeutic outcomes of these patients; and what might be the probability of pregnancy in couples with an UPOR. Therefore, a retrospective study was conducted in patients <35 years who had normal ovarian reserve function and had accepted ART for the first time to address these questions.

## 2 Materials and methods

### 2.1 Inclusion criteria

The inclusion criteria were as follows:

(1) infertile women aged 25–35 years who presented at our center from January 2014 to December 2015;
(2) patients with normal ovarian reserve (follicle-stimulating hormone (FSH) <10 mIU/ml on the second day of the menstrual cycle with a 2- to 9-mm antral follicle count (AFC) of no less than 5^[8,9]^); and
(3) infertile women who first underwent in vitro fertilization and embryo transfer (IVF-ET) or intracytoplasmic sperm injection (ICSI) due to a factor of fallopian tube or a factor of seminal fluid or those with primary infertility had already gone through more than two failed artificial insemination of husband semen.

#### 2.1.1 Exclusion criteria

The exclusion criteria were infertile women with polycystic ovary syndrome or other ovulatory disorders.

### 2.2 Intervention method

All patients were treated according to the standard gonadotrophin stimulation protocol using a short-acting gonadotropin-releasing hormone agonist (GnRH-α, Decapeptyl, Ferring, Switzerland). GnRH-α was administered at 0.1 mg/d from the middle luteal phase of the last menstrual cycle to desensitize the pituitary gland. Recombinant follicle-stimulating hormone (rFSH, Gonal-F, Merck Serono, Switzerland) was given at an initial dose of 150–225 U to promote ovulation when the downregulation criteria were satisfied. Human menopausal gonadotropin (HMG, Livzon Group) was administered at 75–150 U/d based on follicular development, which was monitored by transvaginal B ultrasound (TVS). An intramuscular injection of human chorionic gonadotropin (hCG, Livzon Group) at 5000–10,000 U was administered during the night when the follicles ≥18 mm in diameter accounted for more than half of those ≥14 mm, and the average estradiol level of the follicles ≥14 mm in diameter was not less than 200 pg/ml. Ultrasound-guided puncture was conducted for oocyte retrieval approximately 36 h later. All oocyte retrieval and embryo transplantation surgeries were performed by the same experienced surgeon. The number of transplanted embryos was no more than 3 each time and was generally 2. All frozen embryos were using the vitrification cryopreservation method, and this step was performed by the same embryologist. An intramuscular injection of progesterone (80 mg/d; Zhejiang Xiangju Pharmaceutical Co., Ltd) was given for luteal support.

### 2.3 Grouping

All women were divided into different groups based on the retrieved oocyte number: ≤3, 4–6, 7–9, 10–12, 13–15, 16–18, 19–21, 22–24 and ≥25. The preliminary analysis was based on the clinical pregnancy rate of fresh transfer cycle and the cycle cancellation rate. If it was necessary, the women would be regrouped, and further statistical analysis would be mainly conducted with the reclassified groups.

### 2.4 Observational indexes

The observational indexes were the retrieved oocyte number, clinical pregnancy rate of fresh transfer cycle (number of clinical pregnancies from fresh cycles/number of fresh transfer cycles), fresh cycle cancellation rate (number of canceled fresh transfer cycles/number of initiated cycles), cumulative clinical pregnancy rate (total cases of clinical pregnancy/total cases), cumulative abortion rate (total cases of spontaneous abortion/total cases of clinical pregnancy), cumulative live birth rate (total cases of live birth/total cases), basic Follicle-stimulating hormone (FSH), basic luteinizing hormone (LH), and antral follicle count (AFC).

### 2.5 Determination of observational indexes

Follicle diameter was measured by TVS as the mean of the vertical and horizontal diameters on the maximal surface of the follicle. Hormone levels were determined using chemiluminescence methods.

### 2.6 Statistical analysis

All statistical analyses were conducted using SAS 9.3 statistical software. Non-normally distributed quantitative data are presented as the median (interquartile range) [M(Q)], and groups were compared using the rank sum test. Normally distributed quantitative data are presented as the mean ± standard deviation (X ± SD), and groups were compared by analysis of variance. Rates were compared using the chi-square test. *P* < .05 indicated a statistically significant difference.

The study was approved by the Medical Ethics Committee of Chengdu University of Traditional Chinese Medicine (No. 2011-3).

## 3 Results

### 3.1 Comparison of basic trends

**Figure 1:**
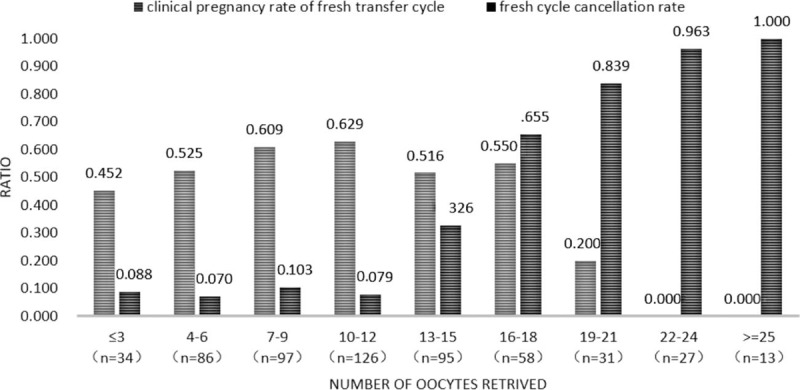
Clinical pregnancy rate in fresh transfer cycles and the cycle cancelation rate.

A total of 567 patients were included in this study. All the patients for the first time underwent assisted reproductive treatment. The average age of patients was 29.99 ± 2.71 years (25–35 years) and the median of Sterile time was 4 (0.33–15) years. In 34 cases, the number of retrieved oocytes was between 0 and 3, accounting for 5.99% of the patients; additionally, in 86 cases, the number of retrieved oocytes was between 4 and 6, accounting for 15.17% of the patients, and in 120 cases, the number of retrieved oocytes was less than 7, accounting for 21.16% of the patients. The number of cases, clinical pregnancy rate of fresh transfer cycles, and the fresh cycle cancellation rate of the remaining groups are shown in Figure 1.

**Figure 2:**
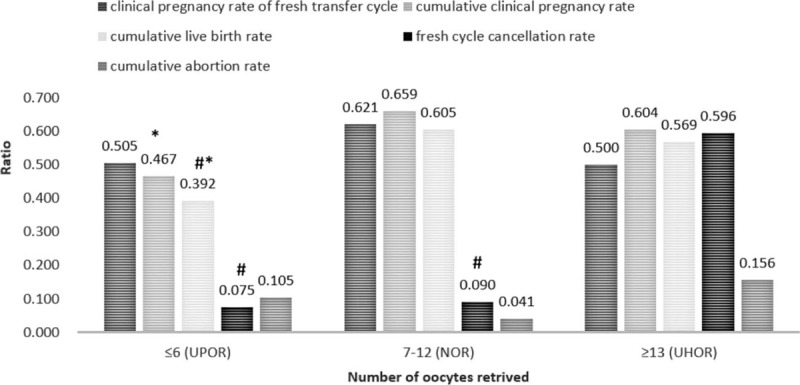
Comparison of therapeutic outcomes in the UPOR, NOR, and UHOR groups. ^∗^, significant difference compared with the NOR group; #, indicates a significant difference compared with the UHOR group. NOR = normal ovarian response, UHOR = unexpected high ovarian response, UPOR = unexpected poor ovarian response.

According to the trends shown in Figure 1, the clinical pregnancy rates were higher than 60% in the groups with 7–9 and with 10–12 retrieved oocytes. However, the clinical pregnancy rate was much less than 60% in the groups for which the number of retrieved oocytes was less than 7. In the groups for which the number of retrieved oocytes was equal to or more than 13, the OHSS risk increased, the cycle cancellation rate increased significantly, and the clinical pregnancy rate was lower than 60%. Therefore, the groups with 7–9 retrieved oocytes and with 10–12 retrieved oocytes were integrated into one group, which was termed the normal ovarian response (NOR) group due to the highest pregnancy rate and lower cycle cancellation rate, with a total of 223 cases. The groups for which the number of retrieved oocytes was less than 7 were also integrated into one group, termed the unexpectedly poor ovarian response (UPOR) group due to the smaller number of retrieved oocytes and the clearly lower clinical pregnancy rates, which were at least decreased by 8% compared with that of the NOR group, with a total of 120 cases. The groups for which the number of retrieved oocytes was greater than 12 were integrated into another group, termed the unexpectedly high ovarian response (UHOR) group for the significantly decreased pregnancy rate, greatly increased cycle cancelation rate due to the high ovarian response, and increased OHSS risk, with a total of 224 cases. Figure 2 shows the clinical pregnancy rate of the fresh transfer cycle, fresh cycle cancellation rate, cumulative clinical pregnancy rate, cumulative abortion rate, and cumulative live birth rate of the three groups after the consolidations. Those groups whose oocyte retrieval numbers bordered those of the NOR and UHOR groups and with similar fresh transfer cycle clinical pregnancy rates were merged into the respective groups.

The fresh transfer cycle clinical pregnancy rate was highest in the NOR group (*P* = .057). The cumulative clinical pregnancy rate and cumulative live birth rate showed the following trend: NOR group>UHOR group>UPOR group (*P* = .001). The fresh cycle cancellation rate showed the following trend: UHOR group>NOR group>UPOR group (*P* < .0001). The cumulative natural abortion rate showed a clear trend of UHOR group>UPOR group>NOR group (*P* = .266).

### 3.2 Comparison of other related factors

After obtaining the abovementioned results, we retrospectively compared the baseline data of the three patient groups. The results showed that although the patients were all <35 years with normal ovarian reserve function, significant differences in the baseline data were observed. A comparison of the results is shown in Table 1.

**Table 1:**
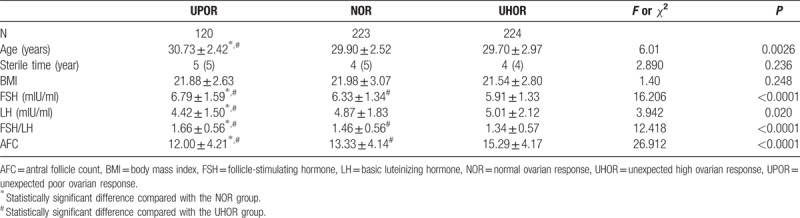
Comparison of baseline parameters among the three groups.

The baseline data comparison among the three groups revealed that age followed the trend of UPOR group>NOR group>UHOR group (*P* = .0026). Differences in the period of infertility and BMI were not significant. Moreover, baseline FSH levels and the FSH/ LH ratio showed the following trend: UPOR group>NOR group>UHOR group (*P* < .0001). The LH level and AFC showed the same trend: UHOR group>NOR group>UPOR group (*P* < .020).

### 3.3 Analysis of factors related to ovarian responsiveness

The multiple regression analysis was carried out to study the relationship between the above basic parameters and the number of retrieved oocytes. The results showed that the main basic parameters related to the number of retrieved oocytes were patient age, basal FSH, basal LH, and AFC. As the definition of poor ovarian response was that the number of retrieved oocytes was no more than 4, only the basic FSH (*χ*^2^ = 3.865, *P* = .0493) and AFC (*χ*^2^ = 16.011, *P* < .0001) were related to the ovarian responsiveness. As the definition of ovarian hypo-response was that number of retrieved oocytes was no more than 7, patient age (*χ*^2^ = 6.177, *P* = .0129), basic FSH (*χ*^2^ = 20.585, *P* < .0001), basic LH (*χ*^2^ = 11.689, *P* = .0006), and AFC (*χ*^2^ = 8.053, *P* = 0.0045) were related to the ovarian responsiveness.

**Figure 3:**
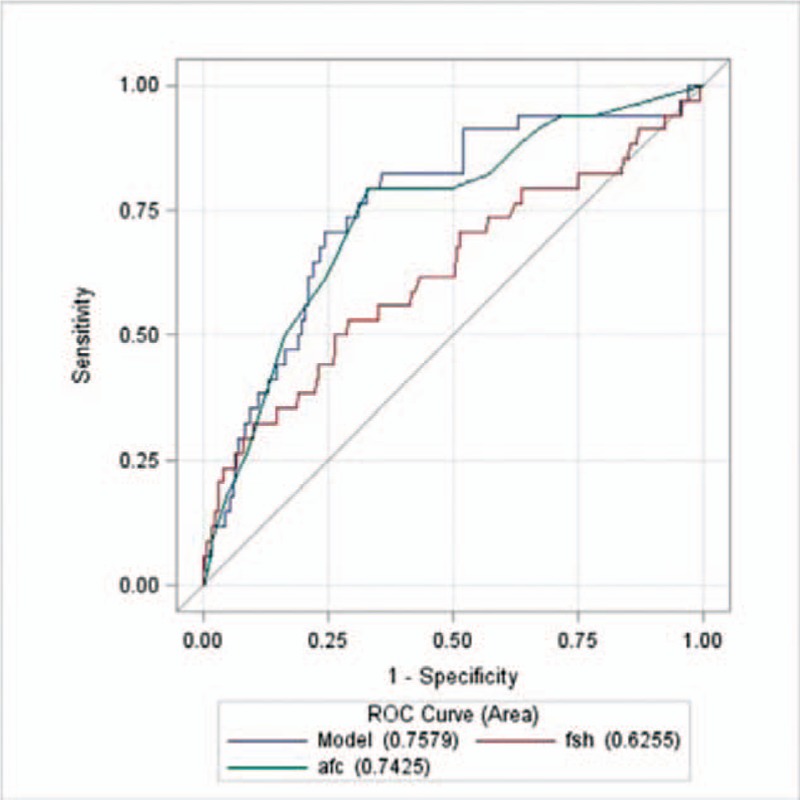
ROC curves for poor ovarian response. *Note*: Model means AFC combined with FSH. AFC = antral follicle count, FSH = follicle-stimulating hormone.

Figure 3 showed that the Area Under Curve (AUC) of AFC alone was 0.7425, that of FSH alone was 0.6255, that of AFC and FSH combined was 0.7579. The predictive value of AFC combined with FSH was better than that of AFC alone (*P* = .0104).

**Figure 4:**
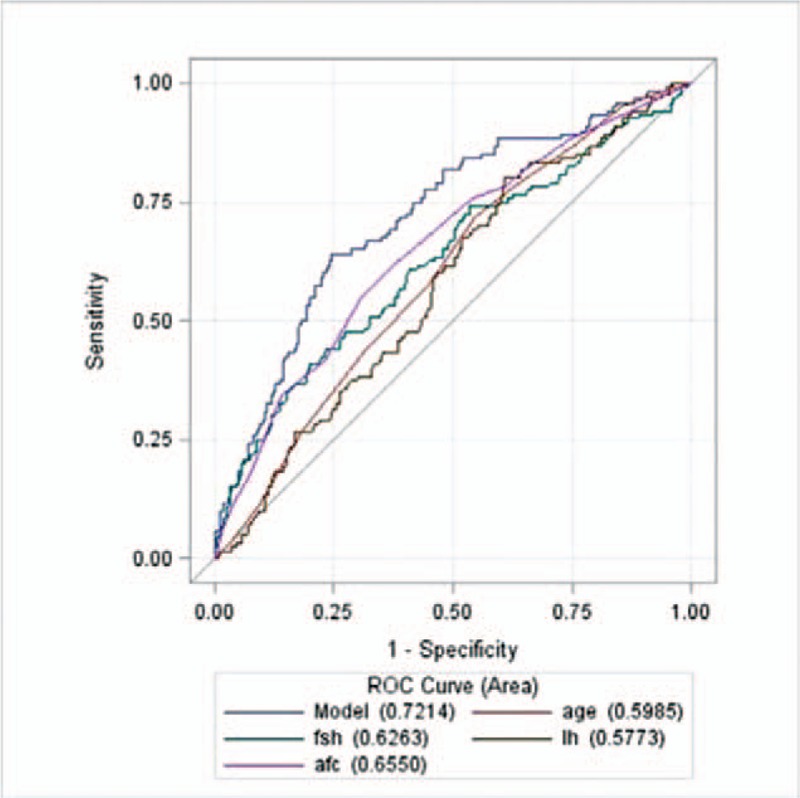
ROC curves for hypo-response. *Note*: Model means AFC combined FSH, AGE and LH. AFC = antral follicle count, FSH = follicle-stimulating hormone.

Figure 4 showed that the AFC had a relatively high predictive value when viewed as a single predictor, with an AUC of 0.6550 (0.5995–0.7104) and the predictive value of four combined indicators (AUC of 0.7214) was better than that of a single predictor (*P* ≤ .001).

## 4 Discussion

This study is the first to report the incidence of unexpected poor ovarian response in women <35 years with normal ovarian reserve function. The results showed that among the Chinese women <35 years with normal ovarian reserve function who accepted standard short-acting long-term treatment at our reproductive center, 21.16% of the patients did not achieve the best treatment outcome due to too few retrieved oocytes, and 5.99% of the patients had an extremely poor treatment outcome.

In the past, the studies about poor ovarian response (POR) were mainly focused on women above >35 years. Because of the ovarian reserve decline associated with age, POR is very common in this population. However, in this study, we found that the incidence of POR in women aged under 35 years is almost as the same as the older ones (the incidence of POR in women >35 years is about 9%–24%) and usually unexpected. This points out that POR is different from DOR; they are two definitions. A young woman with normal ovarian reserve could experience POR either. For the greater probability of success of assisted reproductive treatment in younger women, the high incidence of UPOR must be paid more attention to.

The definition of UPOR in this research was similar to the diagnostic criteria for low prognosis patient in assisted reproductive technology proposed by POSEIDON in 2016.^[4]^ According to the POSEIDON stratification of low prognosis patients, the first group comprises patients <35 years with normal ovarian reserve and a poor ovarian response (less than 4 retrieved oocytes) or a suboptimal ovarian response (a retrieved oocyte number between 4 and 9) to the standard controlled ovarian hyperstimulation treatment. The POSEIDON diagnostic standard of ovarian low prognosis emphasizes the consideration of women with normal ovarian reserve but poor ovarian response. In fact, this group of women should be given more attention than women with decreased ovarian reserve and poor ovarian response. Because physicians have more chances to improve the assisted reproductive treatment outcome of the former group of women, who concurrently are likely to sustain greater psychological pressures than older women, the former group particularly aspires to obtain greater assistive reproductive medical attention.^[10]^ This study is based on such perceptions, and the results support the POSEIDON diagnostic criteria for ovarian low prognosis to a certain degree. However, there are still some nuances between our study and the POSEIDON diagnostic criteria, which will require improvement in future research endeavors. The first difference was between the criteria standards for normal ovarian reserve function. The hormone standard used with the POSEIDON diagnostic criteria is an AMH ≥1.2 ng/ml, whereas the study used an FSH < 10 mIU/ml.^[11]^ The reason for this divergence was that when the included patients underwent IVF treatment, the AMH test had recently been introduced in China and had not been widely used;^[12]^ thus, the FSH test was still being used at this center for this determination. The second difference was in the criteria for the number of retrieved oocytes to define the suboptimal response group. The POSEIDON low ovarian prognosis defined the number of retrieved oocytes as between 4 and 9 as the standard to judge a suboptimal response group. The data from this study showed that when the number of retrieved oocytes was between 4 and 6 for women with normal ovarian reserve function, the assisted reproduction treatment success rate decreased clearly. The differences may be derived from racial differences, age, treatment protocol, and other factors.^[2]^ While POSEIDON proposed definitions of low prognosis patients, the group also indicated that for the diagnostic criteria to be applied in each center, indicators including the 2PN rate and the retrieval rate of mature oocytes at each center might be used to predict how many oocytes might be required from each patient for assisted reproduction treatment success. That is, the POSEIDON diagnostic criteria considered the possibility that various differences among the centers might affect the standard for assessing a low ovarian prognosis. The results of this study also support the existence of such differences.

Evidently, the prognosis of those patients with UPOR might have improved by increasing the initial dose of r-FSH in the subsequent treatment; however, not all patients will have an improved prognosis because the causes of UPOR are not entirely clear. Perhaps such causes are related to polymorphisms of the FSH receptor gene,^[13,14]^ to the polymorphisms of the LH receptor gene,^[15,16]^ or to other unknown factors.

Although the cause of an UPOR may not be known, a poor prognosis may not be completely unpredictable. Our retrospective analysis found that although the patient's age, basal FSH, and the number of AFC were in the normal reserve range, the subtle differences between the different groups had statistical significance. Further regression analysis also found that when the definition of poor ovarian response was that the number of retrieved oocytes was no more than 4, only basal FSH and AFC could be used to predict the ovarian responsiveness, but when the definition of ovarian hypo-response was that the number of retrieved oocytes was no more than 7, age, basal FSH, basal LH, and AFC had predictive value on prognosis of COH treatment. The imaging diagnosis is very important in modern medicine,^[17,18]^ especially for the female diseases.^[19–21]^ In both poor ovarian response group and ovarian hypo-response group, the most powerful single predictive indicator was AFC. However, the most powerful predictive model for UPOR was AFC combined with other indicators, not AFC only. This situation indicates that poor ovarian response may be caused by many factors; DOR is just the most direct reason. So, it is unreasonable to predict ovarian response based on single index in the course of COH treatment, especially only by AFC. Multiple factors should be considered. Moreover, under different diagnostic criteria, the reasons for poor ovarian response varied. Compared with poor ovarian response, the etiology of ovarian hypo-response may be more complicated. In addition, the results of this study suggest that the decline of women's ovarian function may be much earlier than the age we are familiar with. It is known that an age of 35 years, or a basal FSH over 10 mIU/ml, or an FSH/LH ratio greater than 2.5, or a base number of antral follicles less than 5 may be used as the divide for the decline of ovarian function.^[22]^ However, the results of this study showed that ovarian functional decline was perhaps already underway when the women had reached 30 years of age or had a basal FSH of more than 7 mIU/ml, or FSH/LH ratio more than 1.5, or with less than 10–13 ovarian follicles. These findings suggest that when the physician encounters patients with such types of ovarian reserve functions, he or she should consider increasing the initial dose of FSH and supplementing LH during the first round of COH, which might maximally avoid an UPOR.

## 5 Limitation

As mentioned above, the absence of AMH was a limitation of this study. So, we will keep observing this kind of patients to learn the relationship between AMH and UPOR further. Another limitation was the size of sample. It could be noticed obviously that there were only 34 patients in the UPOR group. Compared with the other two groups, the size was quite small. So, similar study with larger size is still needed to test and verify our result.

## 6 Conclusion

In conclusion, the results of this study show that a substantial number of women <35 years with normal ovarian reserve function will be affected by an unexpected poor ovarian response. Although the reason for UPOR is not entirely clear, such a response is not entirely unpredictable. The basic evaluation of ovarian function may no longer be simplified into normal and abnormal ovarian reserve functions; rather, by using a detailed numerical analysis of basic parameter, such as basal FSH and LH levels, the ovarian response to ovulation induction may be predicted to some extent. This approach will significantly benefit women undergoing initial IVF treatment.

## Author contributions

**Conceptualization:** Minghui Zhu.

**Data curation:** Hengli Li, Xiaohong Li, Dongmei Tian, Jing Zhuang.

**Formal analysis:** Xiaohong Li, Jing Zhuang, Dongmei Tian.

**Writing – original draft:** Jing Zhuang, Minghui Zhu.

**Writing – review & editing:** Dan Yang.

## References

[1] Sunkara SK Rittenberg V Raine-Fenning N Association between the number of eggs and live birth in IVF treatment: an analysis of 400 135 treatment cycles. Hum Reprod 2011;7:1768–74. 10.1093/humrep/der106 21558332

[2] Drakopoulos P Blockeel C Stoop D Conventional ovarian stimulation and single embryo transfer for IVF/ICSI. How many oocytes do we need to maximize cumulative live birth rates after utilization of all fresh and frozen embryos? Hum Reprod 2016;31:370–6. 26724797 10.1093/humrep/dev316

[3] La Marca A Minasi MG Sighinolfi G Female age, serum antimüllerian hormone level, and number of oocytes affect the rate and number of euploid blastocysts in in vitro fertilization/intracytoplasmic sperm injection cycles. Fertil Steril 2017;108:777–83. 28987789 10.1016/j.fertnstert.2017.08.029

[4] Humaidan P Alviggi C Fischer R The novel POSEIDON stratification of ‘Low prognosis patients in Assisted Reproductive Technology’ and its proposed marker of successful outcome. F1000Res 2016;5:2911. 28232864 10.12688/f1000research.10382.1 PMC5302217

[5] Ata B Kaplan B Danzer H Array CGH analysis shows that aneuploidy is not related to the number of embryos generated. Reprod Biomed Online 2012;24:614–20. 22503277 10.1016/j.rbmo.2012.02.009

[6] Hendriks DJ te Velde ER Looman CW Expected poor ovarian response in predicting cumulative pregnancy rates: a powerful tool. Reprod Biomed Online 2008;5:727–36. 10.1016/s1472-6483(10)60323-9 18983760

[7] Morin SJ Patounakis G Juneau CR Diminished ovarian reserve and poor response to stimulation in patients <38 years old: a quantitative but not qualitative reduction in performance. Hum Reprod 2018;33:1489–98. 10.1093/humrep/dey238 30010882

[8] Campbell S Goessens L Goswamy R Real-time ultrasonography for determination of ovarian morphology and volume. A possible early screening test for ovarian cancer? Lancet 1982;1:425–6. 6121093 10.1016/s0140-6736(82)91622-1

[9] Ferranretti AP La Marca A Fauster BC ESHRE working group on poor ovarian response definition. ESHRE consensus on the definition of ‘poor response’ to ovarian stimulation for in vitro fertilization: the Bologna criteria. Hum Reprod 2011;7:1616–24. 10.1093/humrep/der092 21505041

[10] Baka S Makrakis E Tzanakaki D Poor responders in IVF: cancellation of a first cycle is not predictive of a subsequent failure. Ann N Y Acad Sci 2006;1092:418–25. 17308168 10.1196/annals.1365.040

[11] Practice Committee of the American Society for Reproductive Medicine. Testing and interpreting measures of ovarian reserve: a committee opinion. Fertil Steril 2015;3:e9–17. 10.1016/j.fertnstert.2014.12.093 25585505

[12] Wu XQ Kong R Tian L A consensus of poor ovarian response [in Chinese]. Reprod Contracept 2015;35:71–9.

[13] Yan Y Gong Z Zhang L Association of follicle-stimulating hormone receptor polymorphisms with ovarian response in Chinese women: a prospective clinical study. PLoS One 2013;10:e78138. 10.1371/journal.pone.0078138 PMC3805513 24167601

[14] Alviggi C Conforti A Caprio F In estimated good prognosis patients could unexpected “hyporesponse” to controlled ovarian stimulation be related to genetic polymorphisms of FSH receptor? Reprod Sci 2016;8:1103–8. 10.1177/1933719116630419 26902430

[15] Alviggi C Humaidan P Ezcurra D Hormonal, functional and genetic biomarkers in controlled ovarian stimulation: tools for matching patients and protocols. Reprod Biol Endocrinol 2012;10:9. 22309877 10.1186/1477-7827-10-9 PMC3299595

[16] De Placido G Alviggi C Mollo A Effects of recombinant LH (rLH) supplementation during controlled ovarian hyperstimulation (COH) in normogonadotrophic women with an initial inadequate response to recombinant FSH (rFSH) after pituitary downregulation. Clin Endocrinol (Oxf) 2004;5:637–43. 10.1111/j.1365-2265.2004.02027.x 15104569

[17] Razek AA Lattif MA Denewer A Assessment of axillary lymph nodes in patients with breast cancer with diffusion-weighted MR imaging in combination with routine and dynamic contrast MR imaging. Breast Cancer 2016;23:525–32. 25763535 10.1007/s12282-015-0598-7

[18] Abdel Razek AAK Routine and advanced diffusion imaging modules of the salivary glands. Neuroimaging Clin N Am 2018;28:245–54. 29622117 10.1016/j.nic.2018.01.010

[19] Razek AA Elhanbly S Eldeak A Transrectal ultrasound in patients with hematospermia. J Ultrasound 2010;13:28–33. 23396895 10.1016/j.jus.2009.09.005 PMC3553209

[20] Abdel Razek AA Gaballa G Denewer A Diffusion weighted MR imaging of the breast. Acad Radiol 2010;17:382–6. 20004597 10.1016/j.acra.2009.10.014

[21] Razek AA Gaballa G Denewer A Invasive ductal carcinoma: correlation of apparent diffusion coefficient value with pathological prognostic factors. NMR Biomed 2010;23:619–23. 20232453 10.1002/nbm.1503

[22] Practice Committee of the American Society for Reproductive Medicine. Testing and interpreting measures of ovarian reserve: a committee opinion. Fertil Steril 2012;6:1407–15. 10.1016/j.fertnstert.2012.09.036 23095141

